# Simultaneous hybrid revascularization for symptomatic lower extremity arterial occlusive disease

**DOI:** 10.3892/etm.2014.1513

**Published:** 2013-08-17

**Authors:** JIN HYUN JOH, SUN-HYUNG JOO, HO-CHUL PARK

**Affiliations:** Department of Surgery, Kyung Hee University Hospital at Gangdong, Kyung Hee University School of Medicine, Seoul 134-727, Republic of Korea

**Keywords:** hybrid, endovascular procedures, revascularization, lower extremity, artery

## Abstract

Multilevel revascularization, using a combination of endovascular and open (hybrid) surgery, is increasingly being used. Hybrid surgery allows complex anatomy to be treated by minimally invasive procedures in medically high risk patients. The aim of the present study was to report a novel hybrid surgery for lesions in the multilevel lower extremity arteries and to evaluate the clinical outcomes. Consecutive patients who presented at a single institution between March 2009 and Feburary 2012 were selected for inclusion in the study. The patients had disabling claudication or critical limb ischemia and underwent treatment for revascularization by open surgery or by a combination of open surgery and endovascular procedure. Retrospective analysis was conducted from a prospectively collected database. All procedures were performed by a vascular surgeon in an operating room. Postoperative surveillance in outpatient clinics was conducted at 3 and 6 months and every 6 months thereafter. A total of 76 patients were included in the study with a mean age of 67.1±11.3 years (range, 42–94 years) and the male to female ratio was 67:9. The most common indication for revascularization was Rutherford category IV (resting pain). The immediate technical success rate of hybrid surgery was 90.5%, with an overall limb salvage rate of 97.4%. The primary patency rates of the hybrid and open groups were 100 and 90.9%, respectively (P=0.441). Therefore, the results of the present study indicate that hybrid surgery is a feasible option for the treatment of multilevel peripheral arterial occlusive disease, showing favorable patency and limb salvage rates. These observations indicate that femoral endarterectomy plays a vital role in hybrid surgery.

## Introduction

Endovascular treatment may be used for relatively simple, short lesions in peripheral arterial occlusive disease (PAOD) whereas open surgery is typically used for long segment lesions. According to the TransAtlantic Society Consensus (TASC) II guidelines, published in 2007 (1), relatively short ‘A’ lesions should be treated by endovascular procedures, whereas relatively long ‘D’ lesions should be treated with open surgery. For ‘B’ lesions, since endovascular methods offer sufficiently good results, this approach is preferred, unless an open revascularization is required for other associated lesions in the same anatomic area. The treatment of ‘C’ lesions with open revascularization procedures produces superior long-term results; therefore, endovascular methods should only be used in patients at high risk for open revascularization.

Multilevel involvement is typically observed in PAOD. A staged approach to multilevel occlusive disease was the standard for numerous years, with balloon angioplasty of the iliac artery followed at an interval by infrainguinal surgery (2,3). This approach was a rational strategy at a time when open and endovascular surgical techniques were only performed in separate settings.

Multilevel revascularization, using a combination of endovascular and open (hybrid) surgeries, was first reported in the early 1990s (4). Hybrid surgery is ideal for multilevel lesions, as it allows for minimally invasive treatment on complex anatomy in medically high-risk patients. Examples of multimodal and multilevel vascular reconstructions are common femoral endarterectomy combined with open iliac artery transluminal angioplasty and stent placement (5,6), or infrainguinal bypass originated distal to an iliac or superficial femoral artery (SFA), percutaneous transluminal angioplasty and stent (7). Usually the procedures are performed simultaneously, although individual patient anatomy plays a part in the decision of whether to perform the two procedures simultaneously or not.

The aim of the present study was to report a novel hybrid surgery for lesions involved in multilevel lower extremity arteries and to evaluate the clinical outcomes.

## Materials and methods

### Patients

Consecutive patients from the Kyung Hee University Hospital at Gangdong (Seoul, Korea) were selected between March 2009 and February 2012. This study was approved by the Institutional Review Board (the Kyung Hee University Hospital at Gangdong). Written informed consent for the procedure was obtained from all the patients or their families. Each patient had disabling claudication or critical limb ischemia (Rutherford category 3 and 4–6, respectively) and underwent revascularization by open surgery (open group) or by a combination of open surgery and an endovascular procedure (hybrid group). The patients were retrospectively analyzed using a prospectively collected database.

Demographic and clinical characteristics of the patients were recorded, including comorbidities (hypertension, diabetes, smoking, coronary artery disease, cerebrovascular disease, chronic obstructive pulmonary disease, hyperlipidemia and chronic renal failure), clinical presentation, imaging studies, procedural details and condition on last follow-up. Preoperatively, all patients underwent computed tomography-angiography (CTA) for the evaluation of lower extremity arterial occlusive lesions. Treatment modality was determined by the vascular surgeon based on the CTA images. Endovascular procedures were performed for lesions of TASC classifications A and B whilst open surgeries were performed for lesions of TASC classification C and D and at lesions of joint portion.

### Surgical procedures

Procedures were performed by a vascular surgeon in an operating room. Open surgeries were performed with standard techniques, whilst hybrid surgeries were performed using the BV Pulsera system (Philips, Andover, MA, USA) for fluoroscopic imaging. In hybrid cases with iliac occlusions, crossing the lesion in a retrograde fashion following common femoral artery (CFA) exposure was attempted. When retrograde access failed, the iliac occlusion was crossed in an antegrade fashion and the guidewire was retrieved through the arteriotomy site during endarterectomy or bypass surgery. Self-expandable stents were preferentially used in iliac artery lesions.

In patients who required a femoral endarterectomy and SFA, the SFA lesion was initially crossed in an antegrade fashion following the exposure of the CFA. Angioplasty and/or stenting and femoral endarterectomy followed. Next, the arteriotomy was closed with standard patch angioplasty using the branch of the ipsilateral great saphenous vein.

Patients typically received 3,000–5,000 units heparin following the placement of a sheath, which was not reversed at the end of the procedure. Following surgery, all patients were administered 75 mg clopidogrel for a minimum of 90 days and 100 mg enteric coated acetyl salicylic acid for the rest of their lives.

### Postoperative surveillance

Technical success for an endovascular procedure was defined as a patent vessel with <30% residual stenosis, following postdilatation with restoration of rapid antegrade perfusion. Postoperative surveillance was performed in outpatient clinics at 3 and 6 months and every 6 months thereafter. Clinical assessments of the femoral and distal pulses, ankle-brachial index (ABI) measurements and duplex scanning or CTA were performed when clinically indicated. Loss of patency was defined as a reduction in the ABI of >0.15 or significant stenosis on duplex scanning or CTA.

### Statistical analysis

Data were analyzed using SPSS 19.0 software (SPSS, Inc., Chicago, IL, USA). Kaplan-Meier analysis was used to compare the primary and secondary patency rates of the groups on an intent-to-treat basis. Continuous variables are presented as mean ± SD. Demographic comparisons were performed using Fisher’s exact test for categorical variables and by Mann-Whitney U test for continuous variables. P<0.05 was considered to indicate a statistically significant difference.

## Results

### Patient demographics

A total of 76 patients were included in the study. Demographic and preoperative characteristics of the patients are shown in Table I. The mean age was 67.1±11.3 years (range, 42–94 years) and the male-to-female ratio was 67:9. The most common indication for revascularization was Rutherford category IV (resting pain) and hypertension was the most common comorbidity. Table II shows demographic and preoperative characteristics of the open and hybrid groups. Age, indication for revascularization and comorbidities were similar between the open and hybrid groups. The follow-up period was significantly longer in the open group compared with that in the hybrid group.

### Hybrid and endovascular surgeries

**Figure 1:**
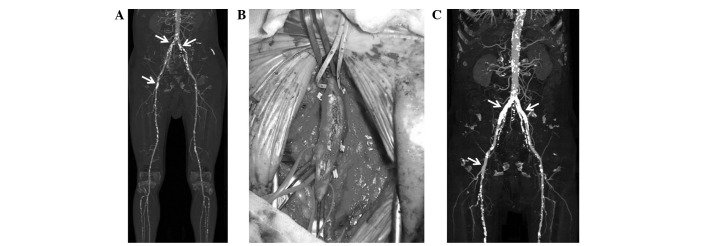
Combined femoral endarterectomy and bilateral iliac kissing stent. (A) Preoperative CTA showing a highly calcified lesion on the right CFA and bilateral common iliac arteries. (B) Image of the right CFA showing calcified plaques which were endarterectomized. (C) Postoperative CTA showing patent iliac stenting and a widely opened right common iliac artery. CTA, computed tomography-angiography; CFA, common femoral artery.

The most common hybrid procedure was a combination of femoral endarterectomy and iliac stenting (Fig. 1). In the case shown, preoperative CTA showed tight, calcified stenosis of the right CFA, as well as bilateral common iliac stenosis (Fig. 1A). Iliac stenting was followed by exposure of the femoral artery. After the endovascular procedure, routine endarterectomy and patch closure was performed (Fig. 1B). Postoperative CTA showed a widely opened right CFA and patent iliac stent (Fig. 1C).

**Figure 2:**
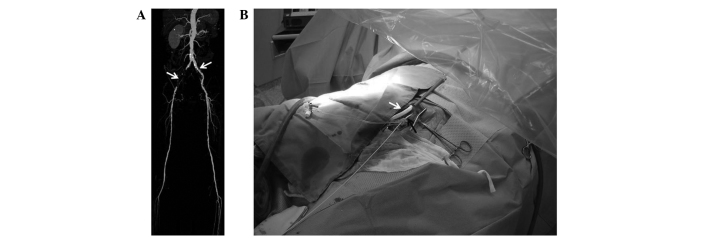
Iliac stenting and crossover femorofemoral bypass. (A) Preoperative CTA showing long segment occlusion of the right iliac artery and focal stenosis of the left common iliac arteries. (B) Image during hybrid surgery showing the artificial graft for a bypass and the vascular introducer sheath for endovascular procedure. CTA, computed tomography-angiography.

**Figure 3:**
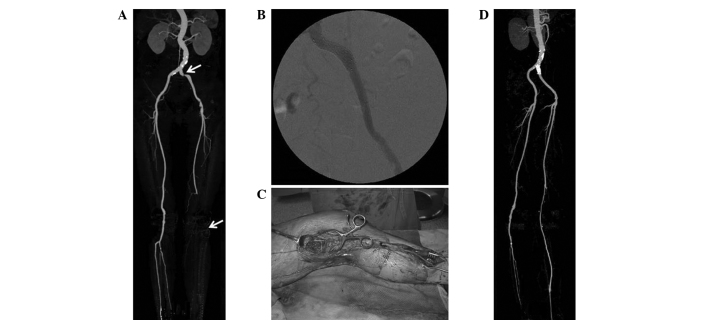
Distal origin bypass and iliac stenting. (A) Preoperative CTA showing long segment occlusion of the left tibio-peroneal artery and tight stenosis of the left common iliac artery. (B) Contrast angiogram of the placed iliac stent. (C) Image of the bypassed vein graft. (D) Postoperative CTA (maximal intensity projection image) of the patent bypassed graft and iliac stent. CTA, computed tomography-angiography.

**Figure 4:**
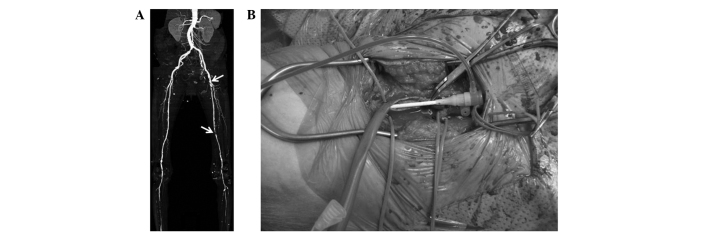
Femoral endarterectomy and endovascular treatment of a lesion in the SFA. (A) Preoperative CTA showing tight stenosis on the left CFA and SFA. (B) Image of the inserted introducer sheath for endovascular treatment of a lesion in the SFA. CTA, computed tomography-angiography; SFA, superficical femoral artery; CFA, common femerol artery.

Another frequently performed hybrid surgery is shown in Fig. 2. Unilateral iliac stenting and crossover femorofemoral bypass was performed in patients with TASC classification A unilateral iliac artery stenosis and TASC classification C or D contralateral iliac occlusion. A typical CTA image is shown in Fig. 2A. For these patients, the bilateral femoral artery was first exposed and tunneling was conducted using a conventional tunneler. Next, the artificial graft was placed in the tunneled portion and intravenous heparin was injected to minimize bleeding. The endovascular procedure followed and finally, bypass surgery was performed. The artificial graft for crossover femorofemoral bypass and the introducer sheath for endovascular procedure is shown in Fig. 2B. Fig. 3 shows an example of the iliac stenting and distally originated bypass used in patients with combined lesions comprising a TASC classification A iliac lesion and C or D lesions below the knee arteries. Fig. 4 shows an example of endarterectomy of the proximal femoral artery and endovascular procedure for TASC classification A or B lesions of the SFA.

Procedural details for hybrid surgery are shown in Table III. The most common open surgery was femoral endarterectomy and patch closure, whilst for endovascular surgery, ipsilateral or bilateral iliac stenting was the most common.

### Success rates

The immediate technical success rate of hybrid surgery was 90.5%. There were two technical failures in the hybrid group. It was not possible to cross over the severely calcified bilateral iliac arteries in one patient, which necessitated unilateral iliac stenting and a crossover femorofemoral bypass. The second patient had a flow-limiting dissection of the SFA during balloon angioplasty, which required bail-out stenting. The ipsilateral ABI increased from 0.53±0.21 to 0.75±0.16 in the hybrid group (P=0.024). This difference was identified to be statistically significant.

**Figure 5:**
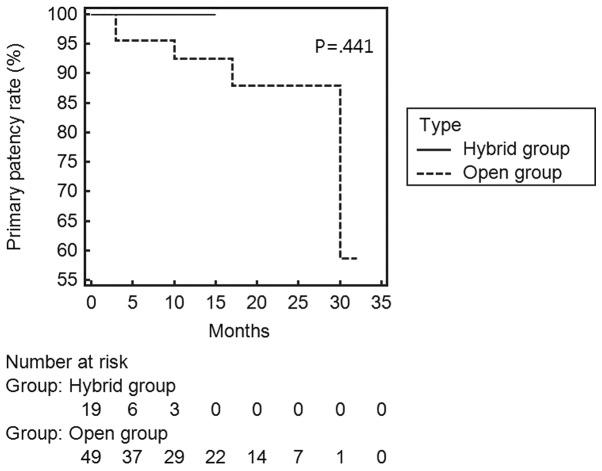
Primary patency rates in patients of the hybrid and open surgery groups. No statistically significant difference was observed between the groups.

**Figure 6:**
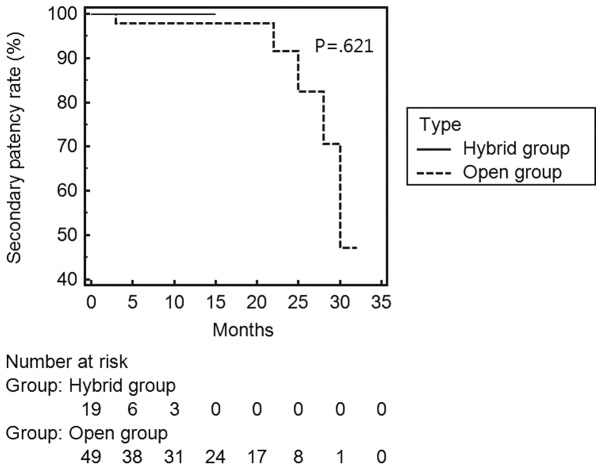
Secondary patency rates in patients of the hybrid and open surgery groups. No statistically significant difference was observed between the groups.

The mean follow-up duration was 10.4±9.4 months (range, 0–32 months) in the two groups and the mortality rate was 6.6%. Three patients succumbed to myocardial infarction, one to overwhelming sepsis and one to intracranial hemorrhage. The overall limb salvage rate was 97.4%. The primary patency rates of the hybrid and open groups are shown in Fig. 5. In the hybrid group, all revascularized arteries were patent during the follow-up period. In the open group, 50/55 (90.9%) were patent at the mean follow-up of 10.4 months; however, the difference between the groups was not statistically significant (P=0.441). Secondary patency rates showed a similar result (Fig. 6).

## Discussion

Combined open and endovascular revascularization may be performed with a staged or simultaneous approach. Endovascular surgery has been enthusiastically embraced by a number of vascular surgeons, leading to increased experience with endovascular interventions. In numerous hospitals, hybrid operating rooms have been constructed where open and endovascular surgery may be performed. It is easy and comfortable to perform simultaneous hybrid surgery in this environment. The hybrid approach has several advantages (8). Firstly, there is no delay in providing complete revascularization to the ischemic limb. Secondly, the length of stay in hospital is reduced and finally, puncture-site complications are eliminated since the target artery is accessed through the surgical field and the access site becomes the location of proximal anastomosis. In the present study, all endovascular procedures were performed through the surgically exposed artery and there were no puncture-site complications in the hybrid group.

When performing a combined femoral endarterectomy and iliac angioplasty/stenting, the procedure sequence may be crucial. Previously, Dosluoglu *et al* (9) recommended pre-arteriotomy guidewire access, where the distal external iliac artery (EIA), CFA, SFA and deep femoral artery are exposed. The puncture site is selected by manually palpating the CFA and EIA. If the CFA is not amenable to puncture due to occlusion or heavy calcification, the distal EIA is punctured. The same technique was employed in the current study. In the conventional percutaneous approach, it may be dangerous to puncture the EIA due to the high risk of retroperitoneal bleeding. However, it is avoided in hybrid surgery since the puncture site may be repaired using an open surgical technique.

In the present study, one patient underwent combined distal origin bypass graft and iliac artery stenting. Distal origin bypass grafts have been shown to have a relatively high limb salvage rate with reasonable morbidity and mortality rates (10,11). The technique of simultaneous SFA endovascular intervention and popliteal to distal bypass was first reviewed by Schneider *et al* (12). In this previous study, 12 patients were treated with SFA angioplasty and distal bypass graft originating from the popliteal artery. There were no perioperative graft failures or amputations and the 2-year primary patency rate was 76%. In the current study, simultaneous common iliac artery (CIA) endovascular intervention and popliteal to distal bypass was performed. This approach may be ideal for this type of combined lesion.

Cotroneo *et al* reported 2-year results of hybrid revascularization (13). The technical success rate was 100% whilst the primary patency rate was 86.2% at 6 months and 79.1% at 24 months. Dosluoglu *et al* stratified hybrid surgery as simple (sHYBRID group) when the endovascular-treated segment was TASC classification A/B and complex (cHYBRID group), when it was C/D (14). The immediate technical success rate was 96% for cHYBRID and 100% for sHYBRID procedures. At the mean follow-up of 30.3 months, the 12- and 36-month primary patency rates in patients who had aortoiliac level interventions in the sHYBRID were 80 and 75%, respectively, and were similar to those in the cHYBRID group, which were 87 and 81%, respectively (P=0.863). Limb salvage rates at 12 and 36 months in patients with critical limb ischemia were similar in the endovascular, sHYBRID and open groups (86 and 80; 94 and 80; and 80 and 74%, respectively); however, these rates were improved in the cHYBRID group (100%; P=0.014). In the present study, the initial technical success rate of hybrid surgery was 90.5%. At the mean follow-up of 10.4 months, the primary patency rates of hybrid and open surgery were 100 and 90.9%, respectively. Hybrid surgery has a theoretical advantage compared with open or endovascular revascularization performed separately, in terms of patency rate. With hybrid surgery, inflow or outflow arteries may be revascularized, which may affect the patency rate. However, as the follow-up period was only 10 months in the current study, a longer-term follow-up is required to confirm this hypothesis.

The annual number of hybrid procedures is increasing. Aho and Venermo (15) reported that this number ranged between 4 in 2004 and 73 in 2011. Of these hybrid procedures, the proportion of endovascular surgeries performed by vascular surgeons increased from 0% in 2004 to 86.3% in 2011. Relatively young vascular surgeons have adopted endovascular surgery and become familiar with it. According to a survey conducted by the Society for Vascular Surgery (16), younger vascular surgeons (those aged <50 years) more frequently reported >50% of their workload being endovascular, compared with older vascular surgeons (aged ≥50 years) (P<0.001). The endovascular skills of vascular surgeons have improved through education and simulator-based endovascular skills training (17–20).

Performing hybrid surgery may greatly reduce hospital charges and the length of stay (LOS). Ebaugh *et al* evaluated the costs of staged versus simultaneous lower extremity arterial hybrid procedures (21). Notably, the unadjusted results showed that hospital charges and LOS more than doubled if staged rather than simultaneous hybrid procedures were performed.

Limitations of the present study include its retrospective nature, and the small number of patients from a single center. The groups were heterogeneous and were not directly comparable. In addition, the follow-up period was too short to fully evaluate the patency rate following hybrid revascularization.

In conclusion, hybrid procedures are a feasible option for multilevel peripheral arterial occlusive disease, with favorable patency and limb salvage rates. The observations of the current study indicate that femoral endarterectomy plays an important role in hybrid surgery.

## Figures and Tables

**Table I:** Patient demographics (n=76).

Characteristics	Patients
Age, years (range)	67.1±11.3 (42–94)
Gender, male:female	67:9
Follow-up, months (range)	10.4±9.4 (0–32)
Clinical severity, n (%)	
Disabling claudication	17 (22.4)
Resting pain	45 (59.2)
Tissue loss	14 (18.4)
Risk factors, n (%)	
Hypertension	52 (68.4)
Diabetes	39 (51.3)
Smoking	27 (35.5)
Coronary artery disease	12 (15.8)
Cerebrovascular disease	11 (14.5)
COPD	6 (7.9)
Hyperlipidemia	4 (5.3)
Chronic renal failure	4 (5.3)

COPD, coronary obstructive pulmonary disease.

**Table II:** Patient demographics (n=76).

Characteristics	Open group (n=55)	Hybrid group (n=21)	P-value
Age, years	65.7±11.8	71.1±8.8	0.076
Gender, male: female	50:5	17:4	
Follow-up, months	13.5±10.1	4.1±4.3	<0.0001
Clinical severity, n (%)			0.479
Disabling claudication	12 (21.8)	5 (23.8)	
Resting pain	35 (63.6)	10 (47.6)	
Tissue loss	8 (14.6)	6 (28.6)	
Risk factors, n (%)			
Hypertension	33 (60.0)	19 (90.5)	0.432
Diabetes	25 (45.5)	14 (66.7)	0.534
Smoking	20 (36.4)	7 (33.3)	0.158
Coronary artery disease	6 (10.9)	6 (28.6)	0.613
Cerebrovascular disease	5 (9.1)	6 (28.6)	0.629
COPD	2 (3.6)	4 (19.0)	0.521
Hyperlipidemia	2 (3.6)	2 (9.5)	0.305
Chronic renal failure	2 (3.6)	2 (9.5)	0.305

COPD, coronary obstructive pulmonary disease.

**Table III:** Open and endovascular procedures in hybrid surgery (n=21).

Type of procedure	Number
Open procedure	
Femoral endarterectomy with patch angioplasty	7*
Femoropopliteal bypass	6
Crossover femorofemoral bypass	5
Femorotibial bypass	3
Popliteo-tibial bypass	1
External-to-internal iliac artery bypass	1
Endovascular procedure	
Ipsilateral iliac stent	15
Bilateral iliac stent	2
Infrainguinal PTA	2
SFA subintimal angioplasty and stent	1
Aneurysm repair using stent-graft	1

* In two patients, bilateral femoral endarterectomy with patch angioplasty was performed.

PTA, percutaneous transluminal angioplasty; SFA, superficial femoral artery.
